# High Spinal Anesthesia Enhances Anti-Inflammatory Responses in Patients Undergoing Coronary Artery Bypass Graft Surgery and Aortic Valve Replacement: Randomized Pilot Study

**DOI:** 10.1371/journal.pone.0149942

**Published:** 2016-03-01

**Authors:** Trevor W. R. Lee, Stephen Kowalski, Kelsey Falk, Doug Maguire, Darren H. Freed, Kent T. HayGlass

**Affiliations:** 1 Department of Anesthesia and Perioperative Medicine, St. Boniface Hospital, University of Manitoba, Winnipeg, Manitoba, Canada; 2 Department of Immunology, University of Manitoba, Winnipeg, Manitoba, Canada; 3 Section of Cardiac Surgery, Department of Surgery, University of Manitoba, Winnipeg, Manitoba, Canada; San Raffaele Scientific Institute, ITALY

## Abstract

**Background:**

Cardiac surgery induces many physiologic changes including major inflammatory and sympathetic nervous system responses. Here, we conducted a single-centre pilot study to generate hypotheses on the potential immune impact of adding high spinal anaesthesia to general anaesthesia during cardiac surgery in adults. We hypothesized that this strategy, previously shown to blunt the sympathetic response and improve pain management, could reduce the undesirable systemic inflammatory responses caused by cardiac surgery.

**Methods:**

This prospective randomized unblinded pilot study was conducted on 14 patients undergoing cardiac surgery for coronary artery bypass grafting and/or aortic valve replacement secondary to severe aortic stenosis. The primary outcome measures examined longitudinally were serum pro-inflammatory (IL-6, IL-1b, CCL2), anti-inflammatory (IL-10, TNF-RII, IL-1Ra), acute phase protein (CRP, PTX3) and cardiovascular risk (sST2) biomarkers.

**Results:**

The kinetics of pro- and anti-inflammatory biomarker was determined following surgery. All pro-inflammatory and acute phase reactant biomarker responses induced by surgical stress were indistinguishable in intensity and duration between control groups and those who also received high spinal anaesthesia. Conversely, IL-10 levels were markedly elevated in both intensity and duration in the group receiving high spinal anesthesia (p = 0.005).

**Conclusions:**

This hypothesis generating pilot study suggests that high spinal anesthesia can alter the net inflammatory response that results from cardiac surgery. In appropriately selected populations, this may add incremental benefit by dampening the net systemic inflammatory response during the week following surgery. Larger population studies, powered to assess immune, physiologic and clinical outcomes in both acute and longer term settings, will be required to better assess potential benefits of incorporating high spinal anesthesia.

**Trial Registration:**

ClinicalTrials.gov NCT00348920

## Introduction

Systemic inflammation has long been recognized as a complication of cardiac surgery [1–4]. Such inflammation is caused by multiple stimuli including tissue injury, contact with the artificial surface of the bypass circuit, hypothermia and reduction of pulmonary blood flow during surgery. Intense inflammatory responses can lead to multiple adverse clinical outcomes affecting the cardiovascular, pulmonary, renal and neurological systems [5–8].

Better understanding of surgically induced systemic inflammatory responses, such as those elicited during cardiopulmonary bypass, is crucial for both prevention and treatment of complications following surgery [9–13]. Demonstration of well-established links between the induction of strong systemic inflammatory responses and subsequent development of poor clinical outcomes in areas as diverse as surgery, sepsis and ischemia have focussed interest on better understanding and modulating acute inflammatory responses. Thus, recent studies of severe acute trauma demonstrate significant changes in leukocyte mRNA abundance of 16,820 out of 20,720 genes examined, some 80% of the human genome—with over 5000 genes exhibiting at least a twofold change compared to healthy controls [14]. Unfortunately, most studies of peri-operative immune changes in humans assess only a very limited panel of biomarkers [2, 4, 12, 13]. Most commonly these include a serum proinflammatory cytokine (ie. IL-6) and/or acute phase protein (ie. C-reactive protein). Essential homeostatic controls on systemic inflammation, such as anti-inflammatory cytokine production, are rarely assessed.

High spinal anesthesia (HSA) using local anesthetics as a supplement to general anesthesia for cardiac surgery continues to generate discussion amongst cardiac anesthesiologists. Although traditionally described as a complication resulting from the high migration or spread of intrathecal anesthesia, there is interest in potential benefits that may accrue from a deliberate application of this approach. When delivered to the appropriate patient, under controlled conditions and with proper technique and monitoring, HSA using local anesthetics has been shown to decrease the stress response to cardiac surgery and cardiopulmonary bypass (CPB). Kowalewski et al. published a case series of patients undergoing Coronary Artery Bypass Graft (CABG) surgery under general anesthesia supplemented with intrathecal local anesthetics who demonstrated excellent hemodynamic stability [15]. Similarly, Lee at al. reported that intraoperative stress responses in CABG patients were decreased with HSA, with these individuals also exhibiting enhanced preservation of beta-receptor function [16]. HSA may also improve aspects of post-operative pain management in patients following either cardiac or non-cardiac surgery [17–19]. We that note a comprehensive meta-analysis of clinical outcome data, specifically focussed on mortality and cardiovascular morbidity, did not indicate benefits from incorporation of spinal analgesia [20]. Similarly, the benefits and limitations of thoracic epidural anesthesia were addressed in a recent metaanalysis [21]. Neither of these studies examined the effects of intrathecal bupivacaine at the doses needed to obtain high spinal anesthesia, or at doses to potentially alter systemic innate immune/inflammatory responses.

Here, we conducted a single-centre, unblinded, pilot study to generate hypotheses on the immune impact of adding high spinal anaesthesia to general anaesthesia during cardiac surgery in adults. A panel of ten well-established and candidate biomarkers characteristic of acute inflammatory responses were examined in over 1100 serum samples to test the hypothesis that the net in vivo inflammatory response elicited during bypass may be ameliorated by addition of HSA to standard general anaesthesia protocols.

## Materials and Methods

### Trial Design

**Fig 1:**
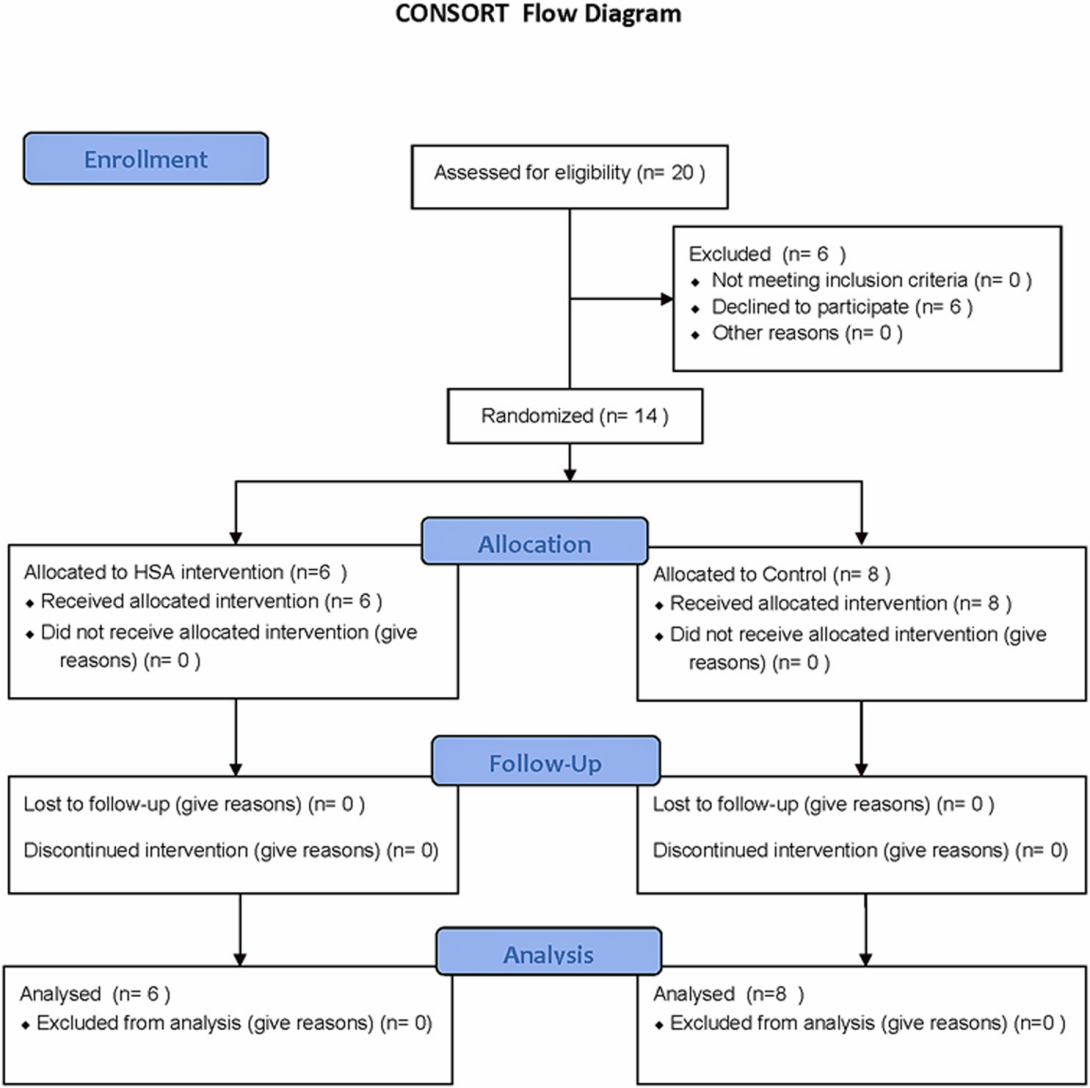
CONSORT flow diagram of the study

This pilot study was a single-centre, unblinded inititive to generate hypotheses on the immune impact of adding HSA to general anaesthesia during cardiac surgery in adults (ClinicalTrials.gov identifier: NCT00348920). Randomization was performed by the study coordinator, via a random number table, into two groups. The intervention group received a high spinal anesthetic (described in detail below) in addition to the standardized general anesthetic received by the control group. The CONSORT flow diagram is presented as Fig 1.

### Patient Population and Ethics Statement

Following approval from the University of Manitoba and St. Boniface Hospital Ethics Committee, written informed consent was obtained from each participant. The study population consisted of fourteen individuals requiring elective or urgent coronary artery bypass graft surgery or elective or urgent surgery for aortic valve replacement for aortic stenosis at St. Boniface Hospital in Winnipeg, Manitoba, Canada. Study recruitment took place from February 2007 to January 2011. Patients undergoing coronary bypass graft surgery or aortic valve replacement surgery for aortic stenosis were targeted for enrollment because these populations were less likely to have contraindications to spinal anesthesia, relative to patients presenting for other types of cardiac surgical procedures. Three patients in each group presented with severe aortic stenosis for aortic valve replacement surgery. All other patients presented with coronary artery disease for coronary artery bypass graft surgery. Exclusion criteria were typical for spinal anesthesia: pre-existing or acquired coagulopathy (INR > 1.4, PTT > 40 seconds, platelet count < 80), intravenous heparin administration or administration of clopidogrel (Plavix) within 10 days of surgery, localized infection or deformity at the site of administration of the spinal, raised intracranial pressure or evolving neurological deficit at surgery, hemodynamic instability requiring pre-operative administration of vasopressors or intra-aortic balloon pump or ventilatory support.

### Perioperative Management and Anesthetic Technique

Prior to surgery, patients received their usual cardiac medications including beta-blockers at the discretion of the attending anesthesiologist. Oral diazepam 0.1 mg/kg, to a maximum of 10 mg, 90 minutes preoperatively was prescribed. All patients had standard monitors applied, including a pre-spinal and pre-induction arterial line, and pulmonary artery catheter. Post-induction of anesthesia monitoring included transesophageal echocardiography. Intraoperative hemodynamic measurements, including pulmonary artery catheter data were measured at sequential time points, including: immediately post insertion of the pulmonary artery catheter, immediately prior to induction of general anesthesia, 10 minutes post tracheal intubation,10 minutes post sternotomy, 20 minutes post sternotomy, 1 minute post separation from CPB, 10 minutes post separation from CPB, and 20 minutes post separation from CPB. Blood samples were drawn for measurement of pro and anti- inflammatory mediators immediately prior to the spinal anesthetic in the group receiving general anaesthetic and HSA or immediately prior to induction in the control general anesthetic only group.

Patients in the HSA group had the spinal administered after insertion of the monitors and pre-induction of general anesthesia. Volume loading consisted of Pentaspan 500 mls intravenously. The patients were then placed in a right lateral decubitus position. Using appropriate sterile technique and a #25 or #22 Whitacre spinal needle, a sub-arachnoid block was administered in a lumbar interspace (L2-3 or L3-4), consisting of spinal bupivacaine 0.75% in dextrose, 6 mls (45mg) and preservative free morphine, 3 mcg/kg (to a maximum of 300 mcg). The patients were then placed supine in a slight head down position (< 5 degrees Trendelenburg), and assessed for the level of the spinal block by testing to cold sensation. After having ensured that a block of at least T-1 was established, general anesthesia was induced using sufentanil (0.0–1.0 mcg/kg), propofol (0.4–2 mg/kg) or pentothal (2–4 mg/kg). The exact doses and drugs were left to the discretion of the attending anesthesiologist. Patients received rocuronium (0.5–1 mg/kg) to facilitate tracheal intubation and the anesthesia will be maintained with sevoflurane (1.0–3.0%) in oxygen, with a minimal end-tidal concentration of 0.8%. The operating room table was leveled 10 minutes post tracheal intubation. Sevoflurane was administered during cardiopulmonary bypass, with a minimum concentration of 1.5% delivered.

Patients in the general anesthesia group received volume loading with Pentaspan 500 mls intravenously and had general anesthesia induced using sufentanil (0.0–1 mcg/kg) and either propofol (0.4–2mg/kg) or pentothal (2–4 mg/kg). The exact drugs and doses were at the discretion of the attending anesthesiologist. Rocuronium (0.5–1 mg/kg) was given to facilitate tracheal intubation. General anesthesia was maintained with sevoflurane (1.0–3.0%) in oxygen, with a minimal end-tidal concentration of 0.8%. Sevoflurane was administered during cardiopulmonary bypass, with a minimum concentration of 1.5% delivered. Ketamine was not administered in either group because its use has been associated with significant decreases in some inflammatory mediators, and may mask the effect of high spinal anesthesia compared to controls.

All patients had the mean arterial pressure kept greater than 60 mmHg with the administration of phenylephrine or ephedrine as required. Glycopyrrolate was given for symptomatic bradycardia (HR<40). Patients received heparin (400 U/kg) to achieve an activated clotting time (ACT) > 500 seconds in order to initiate cardiopulmonary bypass. Tranexamic acid was given as the routine anti-fibrinolytic. The dose of tranexamic acid was 30 mg/kg with a subsequent infusion of 16 mg/kg/hr during the operation.

All patients had standard blood glucose monitoring intraoperatively and regular insulin was administered intravenously as needed to keep the blood glucose levels between 5–8 mmol/L.

At the termination of cardiopulmonary bypass, vasopressor and inotropic agents were administered as needed by the attending anesthesiologist. Milrinone was the first inotropic agent of choice, in addition to phenylephrine for maintenance of mean arterial blood pressure and cardiac output, as required. Protamine was administered as a bolus or infusion of 1 mg/ 100 units of the initial heparin dose and then an infusion of 25 mg/hr was given for 4 hours. Additional boluses of 25 mg were given as needed for ACT> 150 sec or PTT> 40 sec. Post-operative hypertension was treated with intravenous labetolol or intravenous nitroglycerin as deemed appropriate by the attending anesthesiologist. All patients had the standard institutional protocol used for post-operative analgesia. These included parasternal blocks using 0.25% plain bupivacaine (>70 kg patient– 40 mls, >80 kg patient– 50 mls, >90 kg– 60 mls), administered by the surgeon prior to skin closure. Patients also received rectal acetaminophen 1300 mg at the end of the operation. Post-operatively, naproxen 500 mg PR/PO x 1 was administered in the absence of specific contraindications to the use of non-steroidal anti-inflammatory drugs, following usual institutional protocol. Patients were extubated at the end of the operation when hemodynamically stable, in the judgment of the attending anesthesiologist, in the absence of excessive bleeding and when the following criteria were achieved: nasal temperature > 35.5°C, oxygen saturation > 95% with an inspired oxygen concentration of 60% or less, end-tidal CO2 < 55 mmHg while breathing spontaneously, and when the patient was responsive enough to follow simple commands. Patients were transferred to the intensive care unit, as per usual institutional protocol.

### Outcome Measures

Fourteen blood samples were taken through the curse of this study. Individuals were sampled at baseline, multiple time points during surgery, at the end of surgery, 2, 24, 72, 144 hrs post-completion of surgery and, where possible, 28 days post-op. In this hypothesis generating pilot study, the research focus and primary outcome measures were a panel of systemic immune biomarkers and mediators of inflammation. Analyte levels in cryopreserved serum were determined using MesoScale Discovery (MSD, Gaithersburg, MD) electrochemiluminescence assays to quantify binding events on patterned arrays. To provide uniformity in comparing data between different assays, constant internal lab standards (Peprotech, Rocky Hill, NJ and R and D Systems, Minneapolis, MN) were used throughout the study. Briefly, samples were incubated on singleplex MSD plates for 3 h and plates were incubated with detection antibody for 3 h before wash to enhance sensitivity. All other steps were as per manufacturer’s recommendations. Analysis was on a SECTOR™ 2400 instrument (MSD). The operator was blind to the nature of all samples during processing, with subsequent statistical analysis also performed independently. Interassay variation was generally <5–10%. Assays for which MSD plates were not available (pentraxin 3, PTX3 and soluble ST2, sST2) were performed by ultrasensitive ELISA as described [22, 23]. Inter-assay ELISA variability was generally <10%. For some individuals no sample was obtained at certain timepoints or it was consumed before all analyses could be completed.

### Statistics

Immunologic readouts were analysed using Prism 5 software (GraphPad, San Diego, CA). Two-tailed P values, using Wilcoxon Matched Pairs Test for paired analyses comparing individuals’ responses to their own baseline values and Mann Whitney for inter-group comparisions are reported, with differences considered significant if p<0.05.

## Results

The enrollment (Fig 1) and patient characteristics (Table 1) of individuals participating in this randomized pilot identified no significant differences between groups, excepting the mean height of the participants. Three patients in each group presented with severe aortic stenosis for aortic valve replacement surgery. All other patients presented with coronary artery disease for coronary artery bypass graft surgery. Table 2 shows the intraoperative and postoperative patient characteristics. There were no differences seen with respect to pre and on-CPB inotropic support. Four patients in the control group and no patients in the HSA group received post-CPB inotropic support. There were no surgical or anesthetic complications or unintended effects noted in either group.

**Table 1:** Preoperative patient characteristics (mean±SD).

Characteristic	Control (n = 8)	HSA (n = 6)	Significance
Age, yr	61.6±10	66.7±9.9	p = 0.19
Sex (male), n	8	5	p = 0.13
Weight, kg	87.3±12.5	84.8±16.9	p = 0.38
Height, cm	172.9±4.9	164.7±9.7	p = 0.03
BMI, kg/m^2^	29.1±3.3	31.1±4.0	p = 0.17
Current smokers	2	1	NS
Smoking, pack years amongst current smokers	15.38±30	5±12	p = 0.22
Hypertension, n	6	3	p = 0.38
Stroke, n	0	0	NS
Severe lung disease, n	1	0	p = 0.20
Cancer history, n	1	2	p = 0.53
Preop creatinine, mcmol/L	95±24	89±23	p = 0.31
Creatinine > 150 mcmol/L, n	2	1	p = 0.26
Diabetes mellitus, n	2	4	p = 0.70
Canadian Cardiovascular Society (CCS) angina class	2.8±0.9	3.2±0.8	p = 0.19
Preoperative Hgb, g/L	140±222	149±6.2	p = 0.19
Digoxin, n	0	1	p = 0.13
ACE inhibitor, n	1	0	p = 0.20
Nitrates, n	1	2	p = 0.19
Beta-blockers, n	3	4	p = 0.44
Calcium channel blockers, n	0	1	p = 0.13
Diuretic, n	0	1	p = 0.13
Antiarrhythmic, n	0	0	NS

**Table 2:** Intraoperative and postoperative patient characteristics (mean±SD).

Characteristic	Control (n = 8)	HSA (n = 6)	Significance
Pre-op LVEF, %	59.7±12	60.4±9.0	p = 0.46
Pre-CPB phenylephrine, mcg/kg	14.1±13.8	8.4±7.1	p = 0.19
On-CPB phenylephrine, mcg/kg	32.4±29.0	25.3±15.0	p = 0.32
Post-CPB phenylephrine, mcg/kg	17.6±18.9	7.6±9.3	p = 0.16
Post-CPB inotropes, n	4	0	p = 0.02
Duration of surgery, min	254±54	258±77	p = 0.45
Duration of CPB, min	112±26	121±54	p = 0.34
Aortic cross clamp time, min	79±29	89±44	p = 0.32
ICU length of stay, days	1.8±1.2	2.0±2.4	p = 0.43
Total post-op lenth of stay, days	6.2±2.5	5.6±2.6	p = 0.36

### Kinetics of established and candidate immune biomarkers

**Fig 2:**
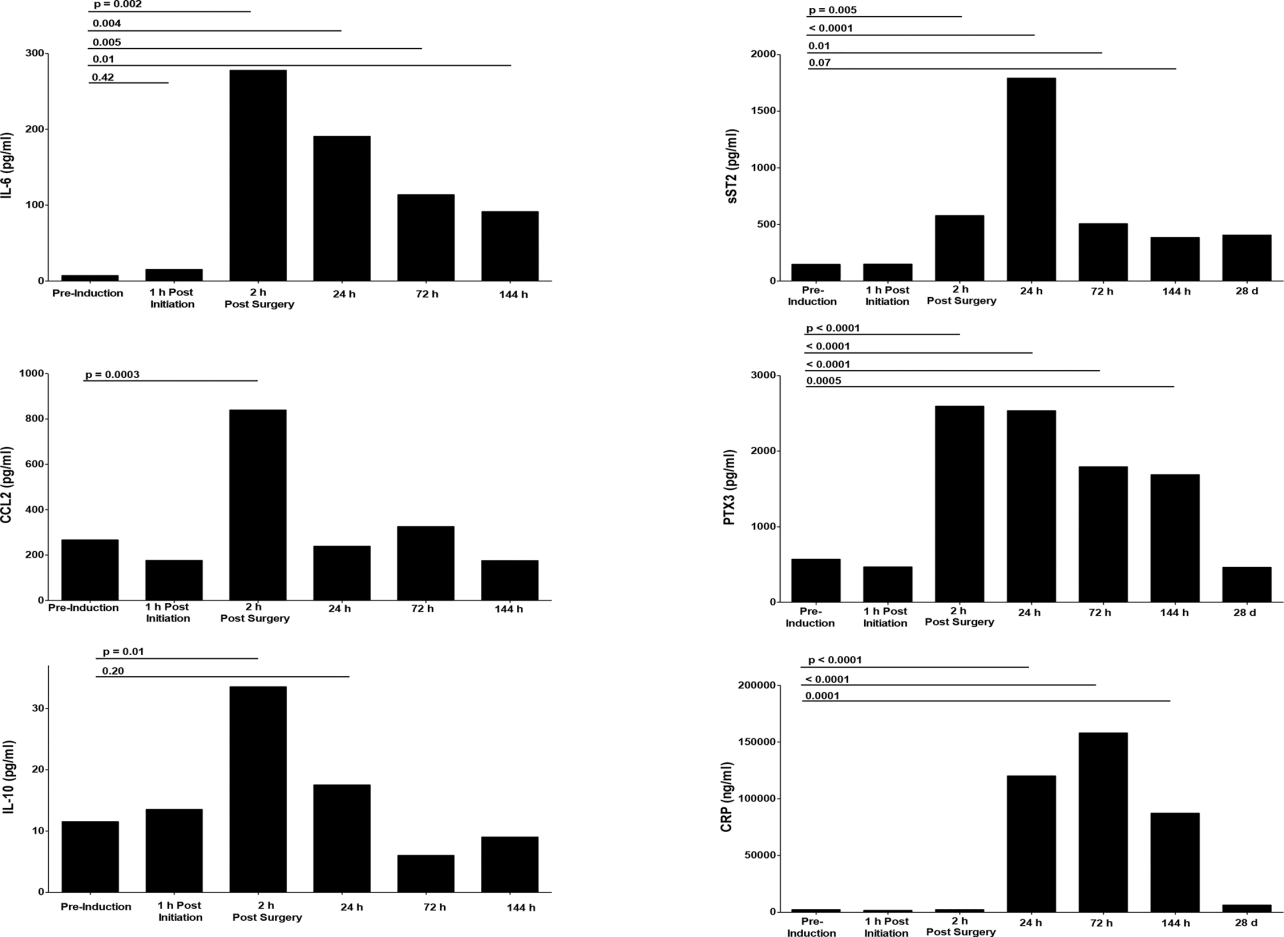
Kinetics of in vivo chemokine, cytokine and acute phase protein responses following CABG. Serum obtained at baseline and the time points indicated was analysed as described at Materials and Methods to assess response kinetics for each biomarker individually. All levels are compared to baseline/study entry conditions. Median responses and statistical significance (Wilcoxon) of longitudinal comparisons relative to pre-induction baseline are shown.

We first determined the kinetics of expression for a panel of well established and candidate pro- and anti-inflammatory cytokine and acute phase protein biomarkers. As is well established, different biomarkers exhibit substantially different kinetics in vivo. For clarity, Fig 2 reports median responses at the most relevant six to seven time points of the 14 samples examined for each biomarker. Except for pre-op values, all times provided are relative to the conclusion of surgery. This time was selected as a common reference point since that was when the acute inflammatory stimuli ended. We note that the mean duration of surgery (Table 2) did not differ between groups and that the variance between individuals within a group is relatively small given the extended kinetics examined. With an average duration of surgery of 4.3 hours, the 2h post-surgery time point occurs approximately 6 hours after the pre-op sample.

As anticipated, IL-6 responses peaked sharply, yielding maximal levels by 2h post-op (Fig 2) and remained significantly elevated 144h post surgery. Chemokine CCL2 (MCP-1), which we hypothesized would offer enhanced sensitivity as an early biomarker of acute inflammatory stimulation, also peaked 2h post-op, but at levels two to four fold higher than those seen for IL-6. Serum IL-1b was without utility as only rare individuals exhibited detectable levels (data not shown, assay sensitivity 2 pg/ml). Among anti-inflammatory responses, increased serum IL-10 levels were first evident two hours post surgery. Among other immune biomarkers of inflammation, soluble ST2, a biomarker of cardiovascular risk and implicated for all-cause mortality [24] as well as acute phase protein pentraxin 3 (PTX3) exhibited strong early and extended responses in vivo. CRP, a classical indicator, peaked considerably later. This kinetic information was used for all analyses presented below, with baseline /pre-operative values shown for each biomarker followed by the time points where in vivo responses were first obvious (as determined in Fig 1).

### Impact of HSA on systemic inflammatory responses: Longitudinal analysis

**Fig 3:**
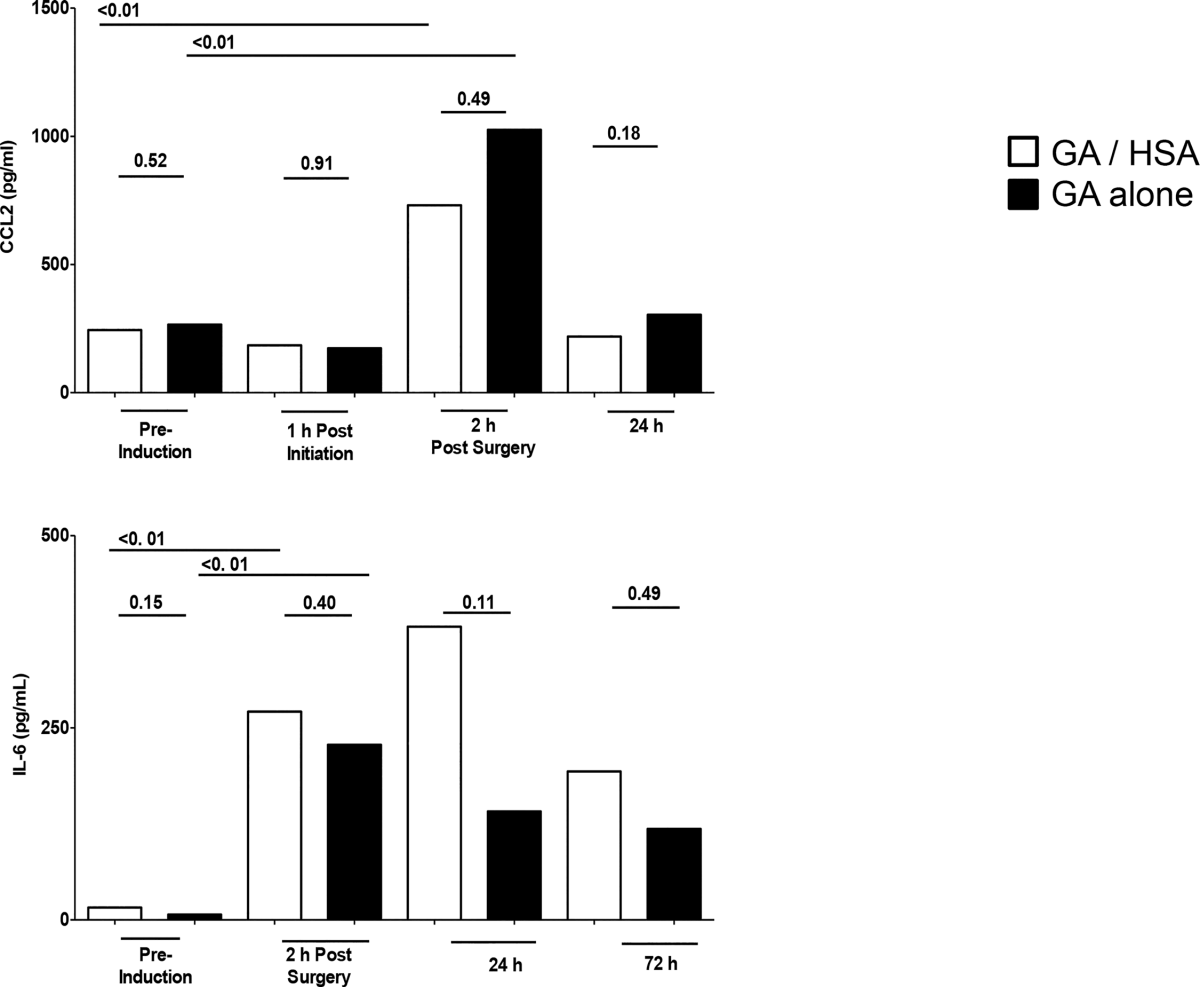
Magnitude and duration of systemic pro-inflammatory cytokine responses in individuals receiving supplemental HSA. Median values are shown for groups receiving HSA plus general anesthetic (open bars) or general anesthetic alone (solid bars). P values reflect inter-group comparisions using Mann-Whitney at the time points indicated.

Having defined the expression kinetics for a panel of pro, anti, and effector inflammatory molecules induced during cardiac surgery, we sought evidence for potential systemic immune changes linked to inclusion of HSA. Baseline pre-surgery serum levels for each immune biomarker were indistinguishable between groups (Fig 3). Because the primary goal was comparison of cytokine responses between GA/HSA and GA alone groups, we focused on the specific time points yielding useful comparisons for each cytokine. With biomarkers exhibiting distinct kinetics (determined in Fig 1), different times were examined for each biomarker.

Surgery led to strong CCL2 responses relative to pre-op, ~ 400% above baseline values, (Wilcoxon p< 0.01 for both groups, Fig 3). Responses were indistinguishable between the HSA and control GA groups. Surgically induced IL-6 responses were also readily evident (Wilcoxon, p<0.01) albeit substantially lower than those of CCL2. They also did not differ detectably between the HSA and control population (Fig 3, Mann-Whitney inter-group comparisons). Collectively these data suggest that inclusion of HSA has no discernable impact on the intensity or duration of systemic pro-inflammatory cytokine responses upon cardiac surgery.

**Fig 4:**
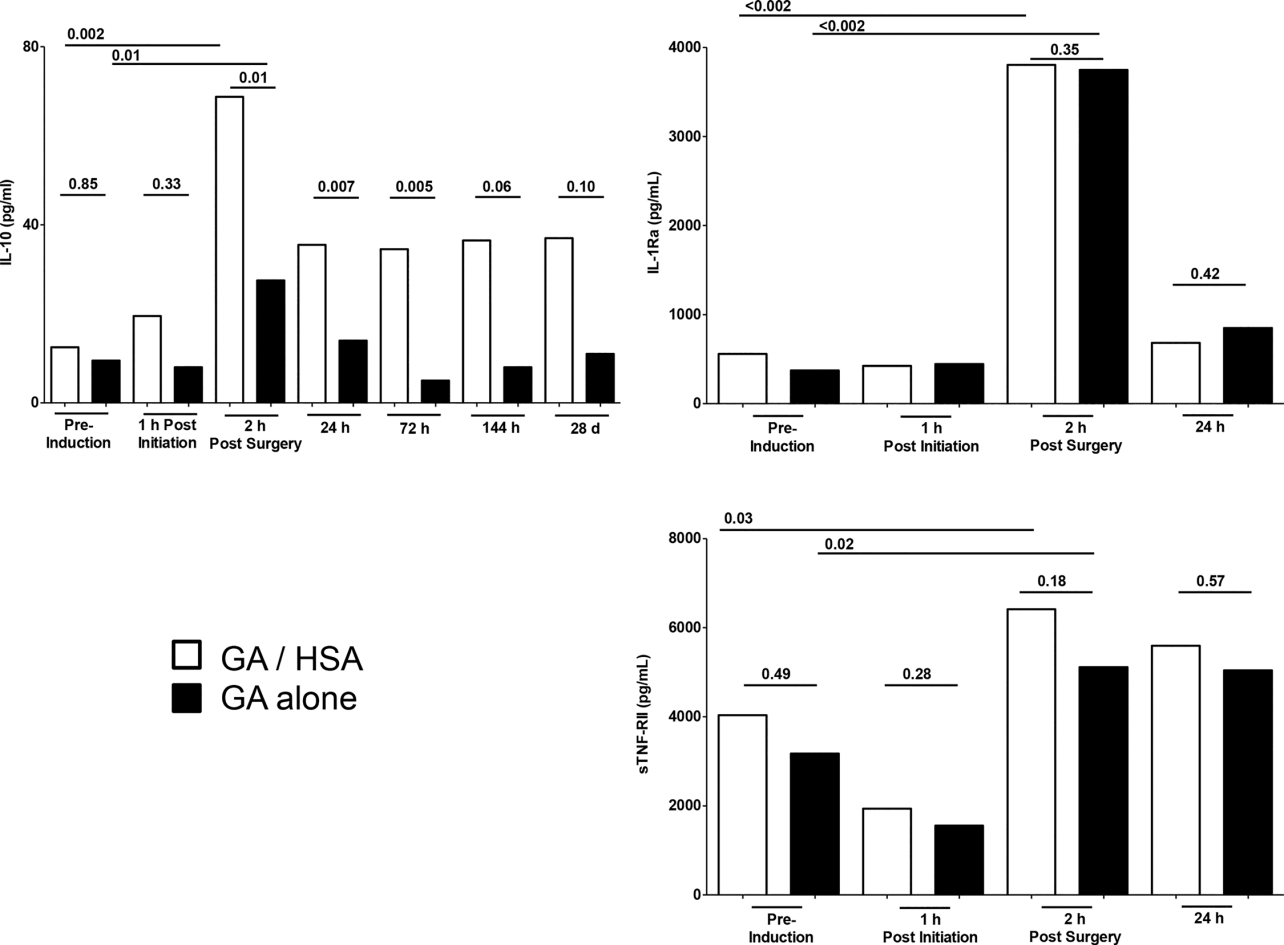
Anti-inflammatory responses of individuals receiving supplemental HSA. Median values are shown for HSA plus general anesthetic (open bars) vs general anesthetic alone (solid bars). P values reflect inter-group Mann Whitney comparisions at the time points indicated.

Anti-inflammatory responses revealed a different picture (Fig 4). Both patient populations generated IL-10 responses upon surgery. The control general anesthesia population exhibited a substantial but transient IL-10 response (Wilcoxon, p<0.01), evident only at 2 hours post conclusion of surgery. The GA/HSA group also exhibited strong IL-10 levels (Wilcoxon, p = 0.002). Intergroup comparisons demonstrated stronger IL-10 responses 2h post surgery (medians 69 vs 27 pg/ml, Mann-Whitney p <0.01). These responses, and differences, sustained at 24h (36 vs 14 pg/ml, p<0.007) and 72h (35 vs 5 pg/ml, p<0.005). A similar, while statistically non-significant, trend to increased IL-10 levels was also evident 144h post surgery (37 vs 8 pg/ml, p = 0.06) in the HSA/GA group. Thus, HSA incorporation was linked to both more intense and prolonged induction of anti-inflammatory IL-10 responses in vivo.

To determine whether multiple anti-inflammatory responses were enhanced, a panel of well–established anti-inflammatory biomarkers were examined. Serum IL-1Ra (Wilcoxon p<0.002) and sTNFRII (p<0.03) responses were induced following surgery. However, the intensity and duration of responses was not different when comparing GA with HSA/GA groups (Fig 4).

**Fig 5:**
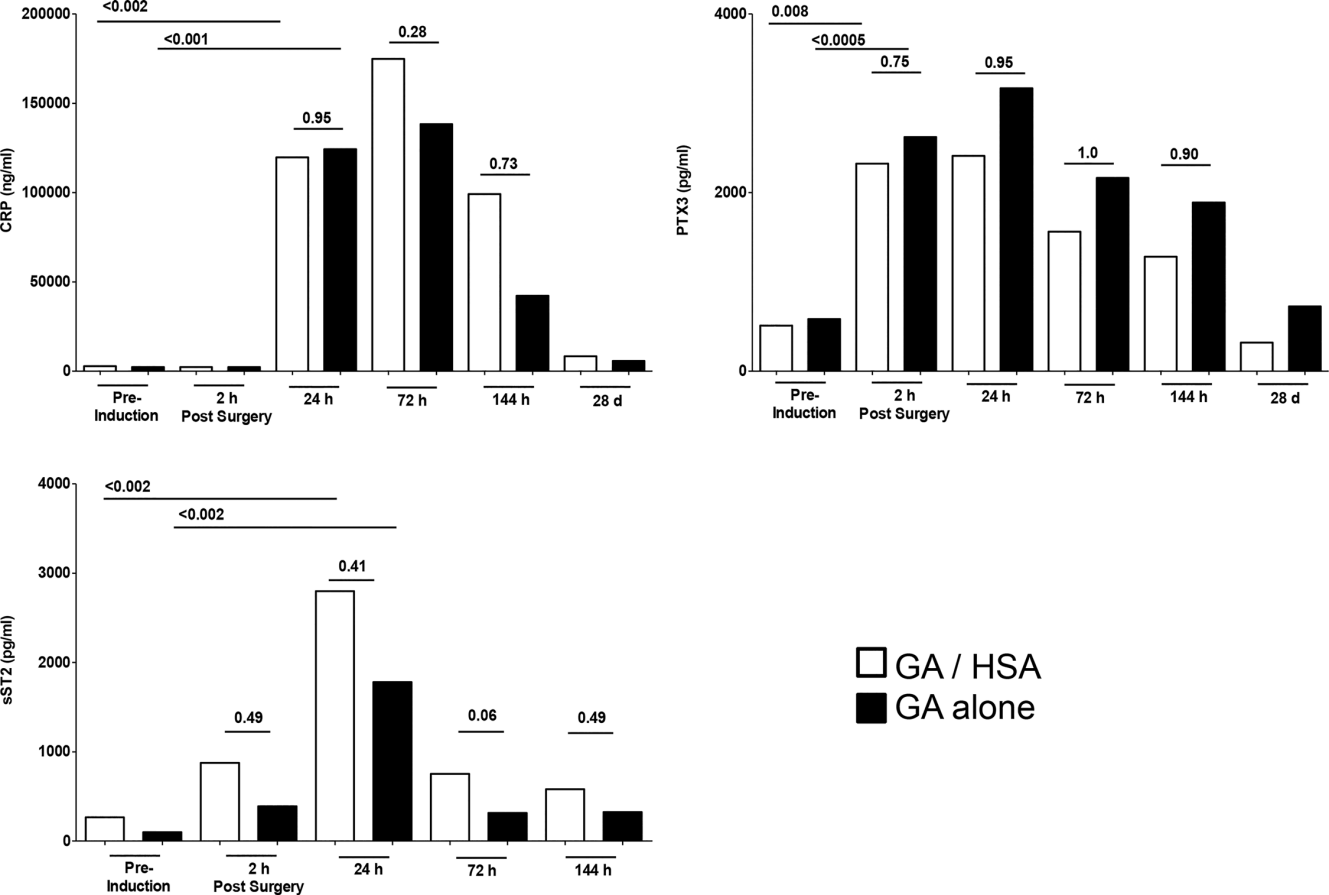
Acute phase protein responses in CABG patients receiving HSA/GA vs general anesthetic alone. Individuals received HSA plus general anesthetic (open) or general anesthetic alone (solid). P values reflect statistical significance (Mann Whitney).

Finally, we compared acute phase protein responses in the two groups. While CRP, PTX3 and sST2 were all strongly induced upon surgery, no evidence was found of an impact of HSA on the intensity or duration of such responses at any time point examined (Fig 5).

## Discussion

Generation of an effective, well balanced inflammatory response is essential for induction of protective immunity. However, surgical interventions, stimuli for which the immune system did not evolve, also induce strong, sometimes prolonged systemic inflammatory responses that can markedly impact clinical outcomes [2,3,4,10,12]. In this hypothesis generating pilot study, we assessed the immunological impact of adding HSA with a non-opiod to standard general anesthesiologic protocols. The specific focus was on potential changes in a broad panel of both established and candidate pro-inflammatory, anti-inflammatory and acute phase protein responses. Pro-inflammatory cytokine production was found to be indistinguishable between HSA and control general anesthetic populations. Conversely, both the intensity and duration of a key anti-inflammatory cytokine response, IL-10, was increased in the population receiving high spinal anesthesia. The data raise the possibility that HSA may be of value in dampening the net systemic inflammatory response that develops during the week following surgery.

While many in vivo human studies of inflammation examine only the arm leading to induction of pro-inflammatory responses (ie IL-6), the data argue that it is equally important to examine endogenous controls on inflammation to understand the net inflammatory response. IL-10 is the best characterized anti-inflammatory cytokine in immune regulation [25, 26]. Produced as a component of both innate and adaptive immune responses, it inhibits inflammation, Th1 and Th2-like activity and multiple immune effector responses. Compelling evidence supports multi-faceted roles for it in modulating both pathogenesis and expression of many human inflammatory disorders. We also note that in a study of over 160 humans encountering blunt force trauma, a condition broadly similar to surgery, the one pathway where gene expression was highly upregulated was IL-10 signalling [14]. Taken together, the increased IL-10 responses seen upon incorporation of HSA in this small, hypothesis generating pilot study suggest potential benefits in reestablishing immune homeostasis in vivo following surgery.

Interestingly, elevated IL-10 production was not accompanied by increases in two independent anti-inflammatory mediators, IL-1R antagonist and sTNF-RII. Whether this reflects a lack of power to identify subtle differences in this pilot or indicates an IL-10 selective impact in enhancement of the host anti-inflammatory response will require further investigation in a larger cohort.

There is longstanding interest in dampening the intensity or duration of surgically elicited innate immune activation. In the 1990's intravenous ketamine was found to reduce the intensity of serum IL-6 responses after cardiopulmonary bypass [5, 27]. Tranexamic acid, a synthetic lysine derivative, was also exhibited beneficial effects on the pro-inflammatory response (assessed solely via IL-6 production) following cardiopulmonary bypass [28]. Other strategies under assessment include dipeptidyl peptidase-4 inhibitors, prednisone and preoperative oral statins [7, 29–31]. We note that most of these studies focus on modulation of pro-inflammatory responses, without assessing changes in anti-inflammatory cytokines. Interestingly, Welters et al., examining intravenous ketamine administration during CPB recently reported an increase in IL-10 levels [32]. This, plus recent reports on the pivotal importance of anti-inflammatory regulators in septic shock and other acute innate inflammatory stimuli [33, 34], reinforce the need to assess in vivo expression of anti-inflammatory as well as pro-inflammatory biomarkers.

Designed as an initial exploration of the impact of HSA on systemic immune responses during and following surgery, this pilot was not intended to, nor sufficiently powered for, assessment of clinical outcomes. We recognize that while excessive inflammation is clearly linked to adverse outcomes in many systems, induction of a more pronounced anti-inflammatory response may or may not translate into significant clinical benefit. Clearly it would be premature to predict a link between altered immune status and clinical success. Confirmation and extension of the finding that the net intensity of inflammatory responses is attenuated in HSA treated populations will require much larger cohorts [4].

A critical issue to be addressed in any translational process is the clinical risk/benefit ratio of adding HSA. Use of spinal anesthesia carries risks in cardiac surgical patient populations, particularly those with critical aortic stenosis. This is due in part to the bradycardia and reduction in systemic vascular resistance that occurs post-administration of high doses of intrathecal anesthetic [35]. We note that the hemodynamic effects of high dose intrathecal bupivacaine, used here, on hemodynamics were previously investigated by Lee et al. [16]. In that study of 38 patients, hemodynamics were stable during the perioperative period for patients undergoing CABG surgery [16]. For patients presenting with severe aortic stenosis for aortic valve replacement, high spinal anesthesia has not previously been studied. Clearly, integration of immune, safety and (putative) clinical benefits from future prospective studies will be required to properly develop a risk/benefit ratio.

It is important to note that a meta-analysis by Zangrillo et al. showed that spinal opioid analgesia (not anesthesia) did not improve outcome measures in cardiac surgical patients [20]. That analysis focused on opiod-driven analgesia and clinical impacts. The current pilot study used a different agent to elicit different effects, (specifically bupivacaine at concentrations needed to obtain high spinal anesthesia), was not intended to seek clinically important outcome differences, but rather to examine possible effects of HSA on systemic immune responses during and following cardiac surgery. Indeed, in comparison to thoracic epidural anesthesia [21] some groups favor the concept of spinal anaesthesia and consider the risk of spinal hematoma remote [36]. This remains an area of ongoing investigation and debate.

In summary, this study demonstrates the importance of examining both arms of immunoregulation of inflammation—pro and anti-inflammatory mediator production upon surgery. The findings of elevated intensity and duration of IL-10 responses upon inclusion of high spinal anesthesia with hyperbaric bupivacaine, taken with prior studies of hemodynamic stability, provides a rationale for future studies to investigate potential clinical benefits when this approach is utilized by cardiac anesthesiologists trained in this specialized technique.

## Supporting Information

S1 CONSORT Checklist S1 CONSORT checklist for High spinal anesthesia: Impact on pro and anti-inflammatory mediators. (DOCX)

S1 Protocol Trial Protocol. (DOCX)
