# Supplementary material for: High Spinal Anesthesia Enhances Anti-Inflammatory Responses in Patients Undergoing Coronary Artery Bypass Graft Surgery and Aortic Valve Replacement: Randomized Pilot Study
Source: PLoS One. 2016 Mar 1;11(3):e0149942. doi: 10.1371/journal.pone.0149942 (PMC4773142; doi:10.1371/journal.pone.0149942)
Supplement: S1 Protocol — (DOCX) [file pone.0149942.s002.docx]

**Supporting Information 1 (S1): Perioperative Management and Anesthetic Technique:**

Prior to surgery, patients received their usual cardiac medications including beta-blockers at the discretion of the attending anesthesiologist. Diazepam 0.1 mg/kg, to a maximum of 10 mg, 90 minutes preoperatively was prescribed. All patients had standard monitors applied, including a pre-spinal and pre-induction arterial line, and pulmonary artery catheter. Post-induction of anesthesia monitoring included transesophageal echocardiography. Intraoperative hemodynamic measurements, including pulmonary artery catheter data were measured at sequential time points, including: immediately post insertion of the pulmonary artery catheter, immediately prior to induction of general anesthesia, 10 minutes post tracheal intubation,10 minutes post sternotomy, 20 minutes post sternotomy, 1 minute post separation from CPB, 10 minutes post separation from CPB, and 20 minutes post separation from CPB. Blood samples were drawn for measurement of stress hormones and inflammatory mediators immediately prior to the spinal anesthetic in the SA group or immediately prior to induction in the control group.

Patients in the high spinal anesthetic (HSA) group had the spinal administered after insertion of the monitors and pre-induction of general anesthesia. Volume loading consisted of Pentaspan 500 mls intravenously. The patients were then placed in a right lateral decubitus position. Using appropriate sterile technique and a #25 or #22 Whitacre spinal needle, a sub-arachnoid block was administered in a lumbar interspace (L2-3 or L3-4), consisting of spinal bupivacaine 0.75% in dextrose, 6 mls (45mg) and preservative free morphine, 3 mcg/kg (to a maximum of 300 mcg). The patients were then placed supine in a slight head down position (< 5 degrees Trendelenburg), and assessed for the level of the spinal block by testing to cold sensation. After having ensured that a block of at least T-1 was established, general anesthesia was induced using sufentanil (0.0 – 1.0 mcg/kg), propofol (0.4 – 2 mg/kg) or pentothal (2-4 mg/kg). The exact doses and drugs were left to the discretion of the attending anesthesiologist. Patients received rocuronium (0.5 – 1 mg/kg) to facilitate tracheal intubation and the anesthesia will be maintained with sevoflurane (1.0 – 3.0%) in oxygen, with a minimal end-tidal concentration of 0.8%. The operating room table was leveled 10 minutes post tracheal intubation. Sevoflurane was administered during cardiopulmonary bypass, with a minimum concentration of 1.5% delivered.

# Patients in the general anesthesia group received volume loading with Pentaspan 500 mls intravenously and had general anesthesia induced using sufentanil (0.0- 1 mcg/kg) and either propofol (0.4 - 2mg/kg) or pentothal (2 - 4 mg/kg). The exact drugs and doses were at the discretion of the attending anesthesiologist. Rocuronium (0.5 – 1 mg/kg) was given to facilitate tracheal intubation. General anesthesia was maintained with sevoflurane (1.0 – 3.0%) in oxygen, with a minimal end-tidal concentration of 0.8%. Sevoflurane was administered during cardiopulmonary bypass, with a minimum concentration of 1.5% delivered. Ketamine was not administered in either group because its use has been associated with significant decreases in some inflammatory mediators, and may mask the effect of high spinal anesthesia compared to controls.

All patients had the mean arterial pressure kept greater than 60 mmHg with the administration of phenylephrine or ephedrine as required. Glycopyrrolate was given for symptomatic bradycardia (HR<40). Patients received heparin (400 U/kg) to achieve an activated clotting time (ACT) > 500 seconds in order to initiate cardiopulmonary bypass. Tranexamic acid was given as the routine anti-fibrinolytic. The dose of tranexamic acid was 30 mg/kg with a subsequent infusion of 16 mg/kg/hr during the operation.

All patients had standard blood glucose monitoring intraoperatively and regular insulin was administered intravenously as needed to keep the blood glucose levels between 5 – 8 mmol/L.

At the termination of cardiopulmonary bypass, vasopressor and inotropic agents were administered as needed by the attending anesthesiologist. Milrinone was the first inotropic agent of choice, in addition to phenylephrine for maintenance of mean arterial blood pressure and cardiac output, as required. Protamine was administered as a bolus or infusion of 1 mg/ 100 units of the initial heparin dose and then an infusion of 25 mg/hr was given for 4 hours. Additional boluses of 25 mg were given as needed for ACT> 150 sec or PTT> 40 sec.

Post-operative hypertension was treated with intravenous labetolol or intravenous nitroglycerin as deemed appropriate by the attending anesthesiologist.

All patients had the standard institutional protocol used for post-operative analgesia. These included parasternal blocks using 0.25% plain bupivacaine (>70 kg patient – 40 mls, >80 kg patient – 50 mls, >90 kg – 60 mls), administered by the surgeon prior to skin closure. Patients also received rectal acetaminophen 1300 mg at the end of the operation. Post-operatively, naproxen 500 mg PR/PO x 1 was administered in the absence of specific contraindications to the use of non-steroidal anti-inflammatory drugs, following usual institutional protocol.

Patients were extubated at the end of the operation when hemodynamically stable, in the judgment of the attending anesthesiologist, in the absence of excessive bleeding and when the following criteria were achieved: nasal temperature > 35.5°C, oxygen saturation > 95% with an inspired oxygen concentration of 60% or less, end-tidal CO2 < 55 mmHg while breathing spontaneously, and when the patient was responsive enough to follow simple commands. Patients were transferred to the intensive care unit, as per usual institutional protocol.
